# Metagenomic Analysis of the Rumen Microbiome of Steers with Wheat-Induced Frothy Bloat

**DOI:** 10.3389/fmicb.2016.00689

**Published:** 2016-05-11

**Authors:** D. W. Pitta, W. E. Pinchak, N. Indugu, B. Vecchiarelli, R. Sinha, J. D. Fulford

**Affiliations:** ^1^Department of Clinical Studies, School of Veterinary Medicine, University of PennsylvaniaKennett Square, PA, USA; ^2^Texas A&M AgriLife ResearchVernon, TX, USA; ^3^Department of Microbiology, Perelman School of Medicine, University of PennsylvaniaKennett Square, PA, USA

**Keywords:** rumen microbiome, frothy bloat, metagenomics, steers, wheat forage

## Abstract

Frothy bloat is a serious metabolic disorder that affects stocker cattle grazing hard red winter wheat forage in the Southern Great Plains causing reduced performance, morbidity, and mortality. We hypothesize that a microbial dysbiosis develops in the rumen microbiome of stocker cattle when grazing on high quality winter wheat pasture that predisposes them to frothy bloat risk. In this study, rumen contents were harvested from six cannulated steers grazing hard red winter wheat (three with bloat score “2” and three with bloat score “0”), extracted for genomic DNA and subjected to 16S rDNA and shotgun sequencing on 454/Roche platform. Approximately 1.5 million reads were sequenced, assembled and assigned for phylogenetic and functional annotations. Bacteria predominated up to 84% of the sequences while archaea contributed to nearly 5% of the sequences. The abundance of archaea was higher in bloated animals (*P* < 0.05) and dominated by *Methanobrevibacter*. Predominant bacterial phyla were Firmicutes (65%), Actinobacteria (13%), Bacteroidetes (10%), and Proteobacteria (6%) across all samples. Genera from Firmicutes such as *Clostridium, Eubacterium*, and *Butyrivibrio* increased (*P* < 0.05) while *Prevotella* from Bacteroidetes decreased in bloated samples. Co-occurrence analysis revealed syntrophic associations between bacteria and archaea in non-bloated samples, however; such interactions faded in bloated samples. Functional annotations of assembled reads to Subsystems database revealed the abundance of several metabolic pathways, with carbohydrate and protein metabolism well represented. Assignment of contigs to CaZy database revealed a greater diversity of Glycosyl Hydrolases dominated by oligosaccharide breaking enzymes (>70%) in non-bloated samples. However, the abundance and diversity of CaZymes were greatly reduced in bloated samples indicating the disruption of carbohydrate metabolism. We conclude that mild to moderate frothy bloat results from tradeoffs both within and between microbial domains due to greater competition for substrates that are of limited availability as a result of biofilm formation.

## Introduction

Grazing winter wheat by beef cattle on the Southern Great Plains of the US is routinely practiced during late fall (November) through spring (late March) each year (Horn et al., 1981; Pinchak et al., 1996; Min et al., 2005, 2006; Horn, 2006; Sij et al., 2007). Vegetative wheat is succulent with a high Crude Protein (CP) value (18–34%) and low Neutral Detergent Fiber (NDF; 30–40%; Horn, 2006). The CP contains a high proportion of soluble protein fraction which undergoes fermentation in the rumen at a rapid rate resulting in the production of an extracellular mucopolysaccharide complex known as biofilm (Min et al., 2005, 2006). Fermentation gases get entrapped in this biofilm with the net result of progressive distention of the rumen, interruption of normal grazing and eructation patterns that culminate in bloat.

Frothy bloat in cattle is a metabolic disorder caused by an array of factors like environment (Majak et al., 1995), structural and chemical composition of forage and animal effects (Clarke and Reid, 1974; Jones and Mangan, 1977; Min et al., 2005, 2006). The onset of bloat is variable between animals and depends on the rate of fermentation of wheat forage and production of ruminal gas, passage rate, and foaming properties of rumen contents (Cole and Boda, 1960; Bartley and Bassette, 1961). Frothy bloat increases the intraruminal pressure with abdominal distension, which interferes with nerve receptors at the reticulorumen juncture and as a result the eructation mechanism is impaired (Cole and Boda, 1960). These adverse effects disturb homeostasis in the rumen, reducing the production value of the animal. About 2–3% mortality is reported due to bloat, and therefore is considered a serious threat to beef farmers (Horn, 2006).

The complex microbiome in the rumen is composed of bacteria, protozoa, and fungi which are primarily responsible for the microbial digestion of plant derived feed resources. The composition of the rumen microbiome dictates the fermentation pathways in the rumen and changes with diet, animal, physiology, and environment (Edwards et al., 2004). The advent of high throughput technologies has enabled us to not only describe the rumen microbiome at a greater resolution than in previous studies, but also allows for linking nutrition and metabolism to the microbiome (McCann et al., 2014; Lima et al., 2015). For example, the ratio of Firmicutes to Bacteroidetes was found to have a positive correlation with milk fat in dairy cows (Jami et al., 2014). Interactions among bacteria, protozoa, and archaea and how these dictate the metabolic phenotype was also demonstrated in lambs (Morgavi et al., 2015). However, information on changes in the rumen microbiome of cattle experiencing bloat is not reported. We have reported that stocker cattle, when transitioned from a hay diet to a winter wheat forage diet, exhibited a significant shift in rumen microbial communities with an increase in Bacteroidetes and reduction in Firmicutes (Pitta et al., 2010). Further, we also found that rumen bacterial populations change with changes in nutritive quality of wheat pasture and that bacterial diversity is tremendously reduced with wheat pasture of higher protein content (Pitta et al., 2014a). It was also reported that bloat in cattle grazing wheat pastures may be caused by an increased production of biofilm, resulting from a diet-influenced switch in the rumen bacterial populations (Min et al., 2006). These findings led us to hypothesize that bloat occurs when the rumen microbial populations are significantly altered due to a change in the fermentation pattern resulting in the formation of biofilm and abdominal distension. In this study we evaluated rumen fluid collected from bloated and non-bloated steers for bloat dynamics, and assessed the changes in the microbial ecology and functional potential of the rumen microbiome with the onset of frothy bloat.

## Materials and methods

### Experimental details

An experiment was conducted during the spring of 2010 (Feb/Mar) with 12 cannulated steers grazing winter wheat on West Walker Ranch, Vernon, TX. All animal surgical, management, and research procedures were conducted under animal use protocols approved by the Texas A&M University Institutional Animal Care and Use Committee. Cannulated steers were checked and visually scored for bloat each morning as per the method of Min et al. (2005). The scoring system was on a 0–3 scale based on the severity of bloat: bloat score BS 0, normal, no visible signs of bloat; BS 1, slight distention of left side of animal; BS 2, marked distention of left side of animal, rumen distended upward toward top of back; BS 3, severe distension, distention is above the top of back and visible from right side of animal, animal has asymmetrical (egg-shape) appearance when walking away.

### Bloated/non-bloated fluid rumen innoculum

The bloat prone period lasted for 2 weeks during late Feb/early Mar 2010. Most animals showed signs of bloat which was scored as BS 1. Having an animal that showed a BS 2 was uncommon. We were able to simultaneously collect rumen contents from three bloated steers (BS 2) and three non-bloated steers (BS 0). Rumen samples from these steers were collected while standing with minimal restraint in an alley. Rumen contents were taken from the lower third of the rumen and ingesta was squeeze filtered through four layers of cheese cloth. Approximately 1.5 L of strained rumen fluid was collected and transferred to bottles immediately to minimize aerobic contamination of samples and archived at −80°C until further analysis.

### Rumen fluid analysis for bloat potential

#### Bloat parameters

Thirty milliliters strained rumen fluid from both bloated and non-bloated rumen contents were poured into a glass cylinder (4.5 cm diameter × 35 cm length) and CO_2_ gas was bubbled through a bottom inlet at 6 Pascal (Pa) for 30 s as a measure of potential foam production. The time required for the foam column to collapse through itself to original fluid volume was used to calculate foam strength. Ethanol-precipitable polysaccharide slime complexes (referred to as bio-film) in clarified rumen fluid were assayed using the method described by Gutierrez et al. (1963). Viscosity in rumen fluid samples was measured using SV-10/SV-100 Vibro Viscometer (A&D Company Ltd., Tokyo, Japan). Viscosity in rumen samples was expressed in mPa.s.

### 16S based bacterial diversity using 454 roche pyrosequencing

The rumen samples were extracted for genomic DNA using the RBBC+C method as described by Yu and Morrison (2004). The extracted DNA was amplified using the bacterial specific primers BSF8 (27F) and BSR357 annealing to the V1V2 region of the 16S bacterial gene. Polymerized chain reaction was performed using Invitrogen's Accuprime Taq DNA polymerase System and the thermal cycling conditions involved an initial denaturing step at 95°C for 5 min followed by 25 cycles (denaturing at 95°C for 30 s, annealing at 56°C for 30 s, extension at 72°C for 90 s) and finally an extension step at 72°C for 8 min as per the method (Wu et al., 2010). The PCR product was then bead purified using Beckman Coulter Agencourt AMPure XP Beads and a magnetic particle concentrator. The amplicons generated for each sample were pooled in equimolar concentration and subjected to pyrosequencing using 454 Junior Roche Platform (GS FLX Titanium).

### 454 Shotgun library preparation

Shotgun sequencing libraries for 454 pyrosequencing were prepared from genomic DNA using the Roche 454 Rapid Library Preparation kit. Samples were fragmented by nebulization using nitrogen gas followed by enzymatic end repair to blunt fragment ends. The 454 specific barcoded adapter sequences were ligated to the blunt fragment ends and the resulting library was simultaneously purified and size selected to remove small fragments using AMPure beads. The final libraries were assessed for quality and concentration on an Agilent 2100 Bioanalyzer using the High Sensitivity assay. Libraries were then pooled in equimolar ratios and used as template for amplification on emulsion PCR (emPCR) using the Roche Lib-L emPCR kit. In the emPCR library fragments were immobilized onto beads which were isolated in an emulsion of oil and PCR reagents to form microreactors allowing for clonal amplification. After emPCR the reactions were purified and enriched for template positive beads which were sequenced on a 454 FLX Sequencer.

### Data analysis

Bloat parameters from bloated and non-bloated samples were analyzed through PROC Mixed procedure of SAS version 9.1. For bacterial diversity comparison between bloated and non-bloated samples, the pyrosequencing reads were analyzed using QIIME pipeline (Caporaso et al., 2010). The raw reads were processed and analyzed using methods described by Pitta et al. (2014b).

For shotgun metagenomic analysis, sequencing reads were assembled using Newbler and the assembled contigs were uploaded to MG-RAST for taxonomy and functional assignments. The raw datasets and processed outputs for all six samples can be accessed at MG-RAST metagenomic project (http://metagenomics.anl.gov/linkin.cgi?project=4727) using the IDs: 4528248.3 (Bloat 1), 4528250.3 (Bloat 2), 4528251.3 (Bloat 3), 4528246.3 (Non-bloat 1), 4528247.3 (Non-bloat 2), and 4528249.3 (Non-bloat 3). Microbial communities in diverse settings have been shown to form syntrophic communities, in which end products from one microbe form the substrate for another (Hoffmann et al., 2013). To identify such associative patterns between and among the most abundant bacterial and archaeal genera, we performed co-occurrence analysis based on Dice index (Dice, 1945). The Dice index was calculated based on the presence and/or absence of the genera using vegan package and visualized using the corrplot package in R (Wei, 2013). Genera were considered present in a sample if its sequence proportion was at least 0.01. The identified phylogenetic and functional groups for the six libraries were downloaded from MG-RAST API and were used for downstream analysis using R software. To test for differences in taxon abundance, a linear model (lm) was constructed with R lm function. Odds ratio was used to identify gene sequences that differentiated bloated and non-bloated rumen samples. To achieve this, each read's taxonomical (phylum level) and functional (Level 1 from SEED database) abundances samples were subjected to a generalized liner model between bloated and non-bloated samples. Further the resultant coefficients and confidence intervals were exponentiated to obtain odds-ratios. For identification of carbohydrate-active gene candidates, protein sequences from this dataset were uploaded in pfam HMM based annotation of CAT (CAZyme Analysis Toolkit) tool available at Carbohydrate Active Enzyme (CAZy) database (Cantarel et al., 2009).

## Results

The bloat prone period occurred for only 2 weeks during early spring in 2010 (this study) while normally the bloat prone period is 3–6 weeks in duration. Precipitation on the West Walker Research Unit and the total protein concentration of wheat pasture were higher in the bloat prone period than in the pre or post bloat prone period (Table 1). The CP concentrations of wheat pasture ranged between 18 and 23% during the pre and bloat prone period.

**Table 1:** **Details of Crude Protein (%) on as fed basis and amount of precipitation (inches) received in pre, during and post bloat prone periods at West Walker Ranch, Vernon**.

**Date of sample collection**	**Bloating period**	**Crude protein (%) in fresh sample**	**Precipitation (inches)**
01/22/10	Pre bloat prone	23.32	0.11^a^
02/18/10	Bloat prone	18.85	4.13^b^
03/05/10	Bloat prone	23.81	1.64^c^
03/22/10	Post bloat prone	14.07	0.94^d^

a Precipitation occurred from 01/20/10 to 01/22/10.

b Precipitation occurred from 01/23/10 to 02/18/10.

c Precipitation occurred from 02/V19/10 to 03/05/10.

d Precipitation occurred from 03/06/10 to 03/22/10.

In this experiment, moderate bloat (BS 2) occurred seldom; hence, we were only able to collect frothy bloated contents on 1 day from three steers which had a bloat score of 2. Three non-bloated steers were simultaneously sampled for rumen contents to serve as controls. Both bloated and non-bloated rumen contents were evaluated for their fermentative and bloating properties (Table 2). Bloated rumen contents had higher pH (6.16 vs. 5.80) than non-bloated rumen contents. Foam strength and viscosity in bloated rumen contents was almost double to that of normal rumen contents (*P* < 0.05). Ethanol precipitated biofilm content was higher in bloated compared to control animal rumen contents (*P* < 0.05).

**Table 2:** **Characteristics of bloated and non-bloated rumen contents**.

	**Bloated**	**Non-bloated**	**SEM**	***P*-value**
pH	6.16	5.80	0.149	0.15
Biofilm	4.36	4.07	0.040	0.007
Foam height	44.44	39.81	1.635	0.11
Foam strength	54.81	29.15	6.032	0.03
Viscosity	4.60	2.07	0.252	0.002

### 16S based rumen bacterial diversity associated with bloat

Approximately 50,000 reads were analyzed from the six bacterial communities with an average of 8000 reads per sample. About 3952 Operational Taxonomic Units (OTUs) were produced by clustering at 97% sequence similarity. Representative sequences from the OTUs were assigned to 19 bacterial phyla.

The most abundant phyla were Firmicutes and Bacteroidetes, which together constituted over 95% of each sample in the study (Table S1 and Figure S1). Among the Firmicutes; Ruminococcaceae, Lachnospiraceae, Erysipelotrichaceae, Clostridiales, and *Incertae Sedis XIII* dominated while Bacteroidetes was dominated by *Prevotellaceae*. The number of genera identified in this study was 135. Considering the most abundant genera, i.e. above 1% relative abundance (Data not shown), only 42% of the total genera were abundant. Members of *Mycobacteriaceae*, an unclassified family in *Actinobacteria, Flavobacteriaceae*, and *alpha proteobacteriaceae* were detected only in the bloated group. The genera that differed between these groups are presented in Tables S2, S3.

### Shotgun metagenomics

In this study, six metagenomic libraries, three each from bloated and non-bloated steers, were constructed with over 200,000 reads per sample results in contigs that ranged between 148,395 and 350,764 (Table 3). The aligned reads were annotated for phylogenetic assignments, functional gene content and CAZymes. Approximately 85% of the assembled reads were annotated for phylogenetic assignments whereas only 30% of contigs were aligned to functional genes of Subsystems database.

**Table 3:** **Sequences information of bloat and non-bloat samples**.

**MGRAST ID**	**Metagenome name**	**No. of contigs**	**PostQC**	**Sequence Lengt (mean ± sd)**	**Archaea (%)**	**Bacteria (%)**	**Identified functional categories**
4529248.3	Bloat_1	201,746	160,850	392 ± 94	8181 (4)	158675 (80)	52,898 (32)
4529250.3	Bloat_2	332,219	260,354	401 ± 90	8786 (2)	263163 (81)	78,603 (30)
4529251.3	Bloat_3	294,687	230,363	402 ± 89	6315 (2)	242423 (81)	72,263 (31)
4529246.3	Non-bloat_1	185,769	148,395	389 ± 93	5097 (2)	150278 (82)	47,136 (31)
4529247.3	Non-bloat_2	449,020	350,764	401 ± 88	7573 (1)	391424 (82)	111,497 (31)
4529249.3	Non-bloat_3	393,054	307,010	397 ± 90	6500 (1)	376043 (84)	99, 280 (32)

### Changes in the rumen microbial ecology with incidence of bloat

The rumen microbial communities were predominated by bacteria (>80%), while archaea accounted for 1–4%, eukarya accounted for <1% abundance (Table S4). Although there appears to be variation among bloated animals, overall archaeal populations were noted to be higher (*P* < 0.01) in bloated samples compared to non-bloated samples. In contrast, the bacterial domain was higher (*P* < 0.01) in non-bloated samples compared to bloated samples. A large proportion (15%) of the sequences was identified as “unassigned” and probably denote novel sequences.

**Figure 1:**
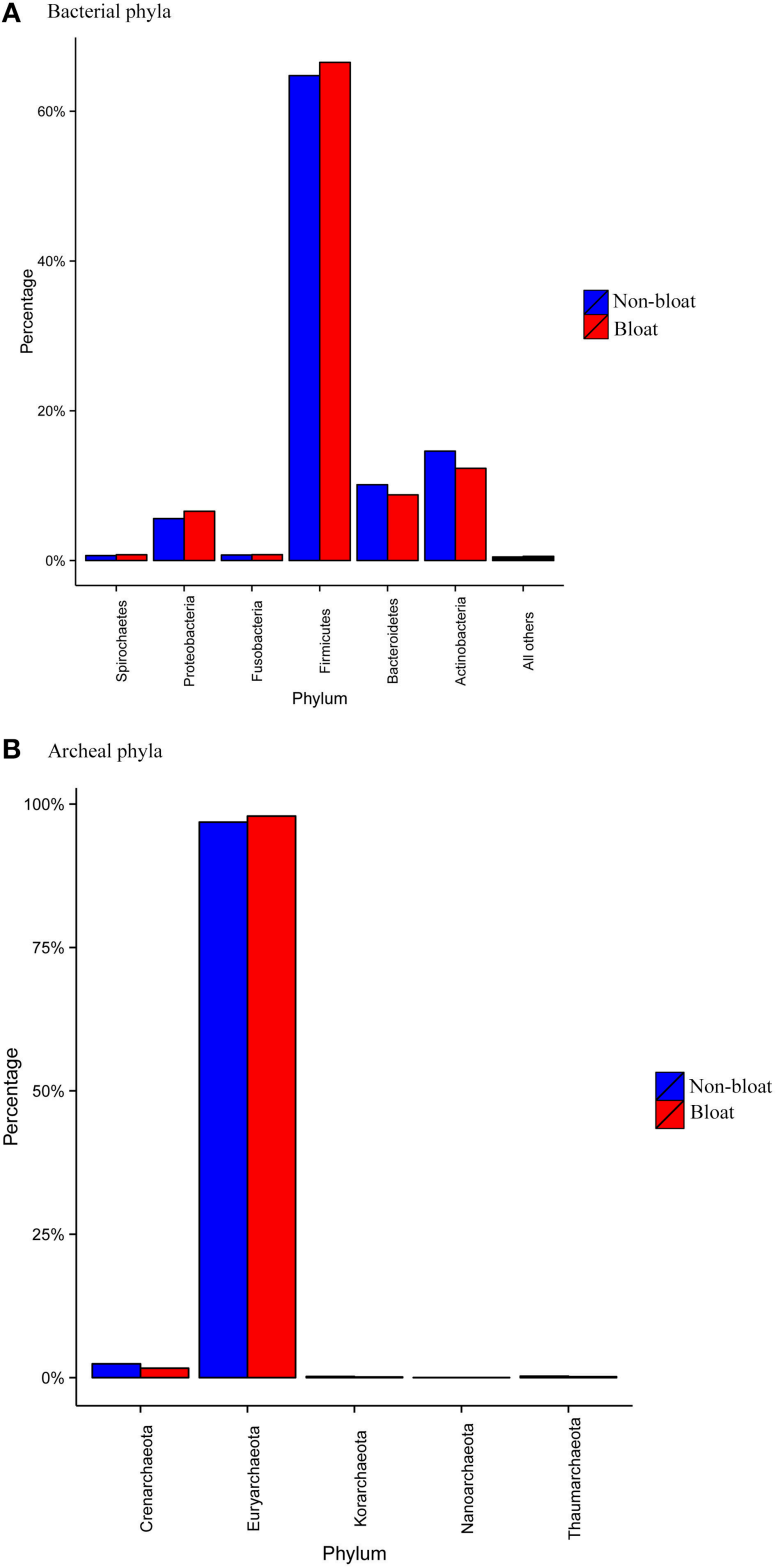
**Mean abundance values (%) of the most abundant (A) bacterial phyla (B) archaeal phyla among bloated and non-bloated rumen contents**.

Within the bacterial domain, about 27 phyla were identified (Table S5). The most abundant phyla across all six metagenomic libraries were Firmicutes (65%) followed by Actinobacteria (15%), Bacteroidetes (10%), and Proteobacteria (5–6%; Figure 1A). The remaining phyla account to <1% abundance respectively. However, the abundance values of a majority of bacterial phyla were found to be different (*P* < 0.01) between bloated and non-bloated samples. Particularly, Firmicutes and Proteobacteria were higher (*P* < 0.01) while the Bacteroidetes and Actinobacteria were lower (*P* < 0.01) in bloated samples compared to non-bloated samples. For the archaeal diversity, Euryachareota alone represented more than 95% of abundance at the phylum level (Figure 1B).

At the genus level (Table 4; Table S6), tradeoffs occurred within the lineages of each bacterial phyla. For example, *Clostridium, Eubacterium*, and *Butyrivibrio* from the Firmicutes group were found to be higher (*P* < 0.01) while *Ruminococcus* was lower in bloated samples when compared to non-bloated samples. Among the Bacteroidetes, *Prevotella* was nearly halved in bloated samples as opposed to non-bloated samples while genera such as *Bacteroides* and *Parabacteroides* increased (*P* < 0.05) in bloated samples. Notably, a majority of genera from Actinobacteria such as *Slackia, Atopobium, Eggerthella, Olsenella, Bifidobacterium, Collinsella, Gordonibacter*, and *Cryptobacterium* were much lower in abundance in bloated samples compared to non-bloated samples. On the contrary, members of Proteobacteria such as *Geobacter, Desulfovibrio, Burkholderia, Pseudomonas, Shewanella, Vibrio, Pelobacter*, and *Escherchia* were more (*P* < 0.05) abundant in bloated samples over non-bloated samples. Among the archaea (Table S7), although genera from Euryarchaeota were highly abundant, tradeoffs occurred among these genera. *Methanobrevibacter* accounted for 52–62% abundance across all samples but was greater in bloated samples while *Methanosphaera, Methanosarchina, Methanocorpusculum, Methanococcus*, and *Methanococcoides* were more abundant in non-bloated samples although their % contribution is small.

**Table 4:** **Mean abundance values (%) of bacterial genera (from the top five phyla) that were significantly different between bloated and non-bloated rumen contents**.

	**Bloat**	**Non-bloat**		**Bloat**	**Non-bloat**
**Actinobacteria**			**Firmicutes**		
Atopobium	2.11	2.81	Abiotrophia	0.50	0.44
Collinsella	0.71	0.91	Alkaliphilus	0.82	0.75
Cryptobacterium	0.42	0.52	Anaerostipes	0.29	0.26
Eggerthella	1.62	1.94	Blautia	2.91	2.62
Frankia	0.10	0.08	Bulleidia	0.21	0.32
Gordonibacter	0.53	0.67	Butyrivibrio	4.38	3.73
Olsenella	1.46	2.05	Catenibacterium	0.30	1.08
Slackia	2.74	3.03	Cellulosilyticum	0.22	0.17
Streptomyces	0.26	0.22	Clostridium	13.41	12.55
**Bacteroidetes**			Coprobacillus	0.20	0.46
Alistipes	0.31	0.13	Coprococcus	1.35	1.27
Flavobacterium	0.13	0.08	Ethanoligenens	0.48	0.51
Paludibacter	0.11	0.07	Eubacterium	7.68	7.13
Parabacteroides	0.60	0.41	Finegoldia	0.15	0.18
Pedobacter	0.13	0.06	Holdemania	0.87	1.07
Porphyromonas	0.24	0.12	Lactobacillus	0.98	1.13
Prevotella	3.58	6.32	Oribacterium	0.80	0.71
Chlorobium	0.12	0.09	Paenibacillus	0.50	0.42
**Fibrobacteres**			Pseudoflavonifractor	1.15	1.17
Fibrobacter	0.35	0.31	Roseburia	1.56	1.39
**Proteobacteria**			Ruminococcus	5.80	6.15
Burkholderia	0.20	0.17	Selenomonas	0.36	0.30
Desulfovibrio	0.27	0.21	Solobacterium	0.35	0.53
Geobacter	0.28	0.25	Subdoligranulum	0.61	0.55
Pseudomonas	0.19	0.17			

### Co-occurence patterns among bacteria and archaea

**Figure 2:**
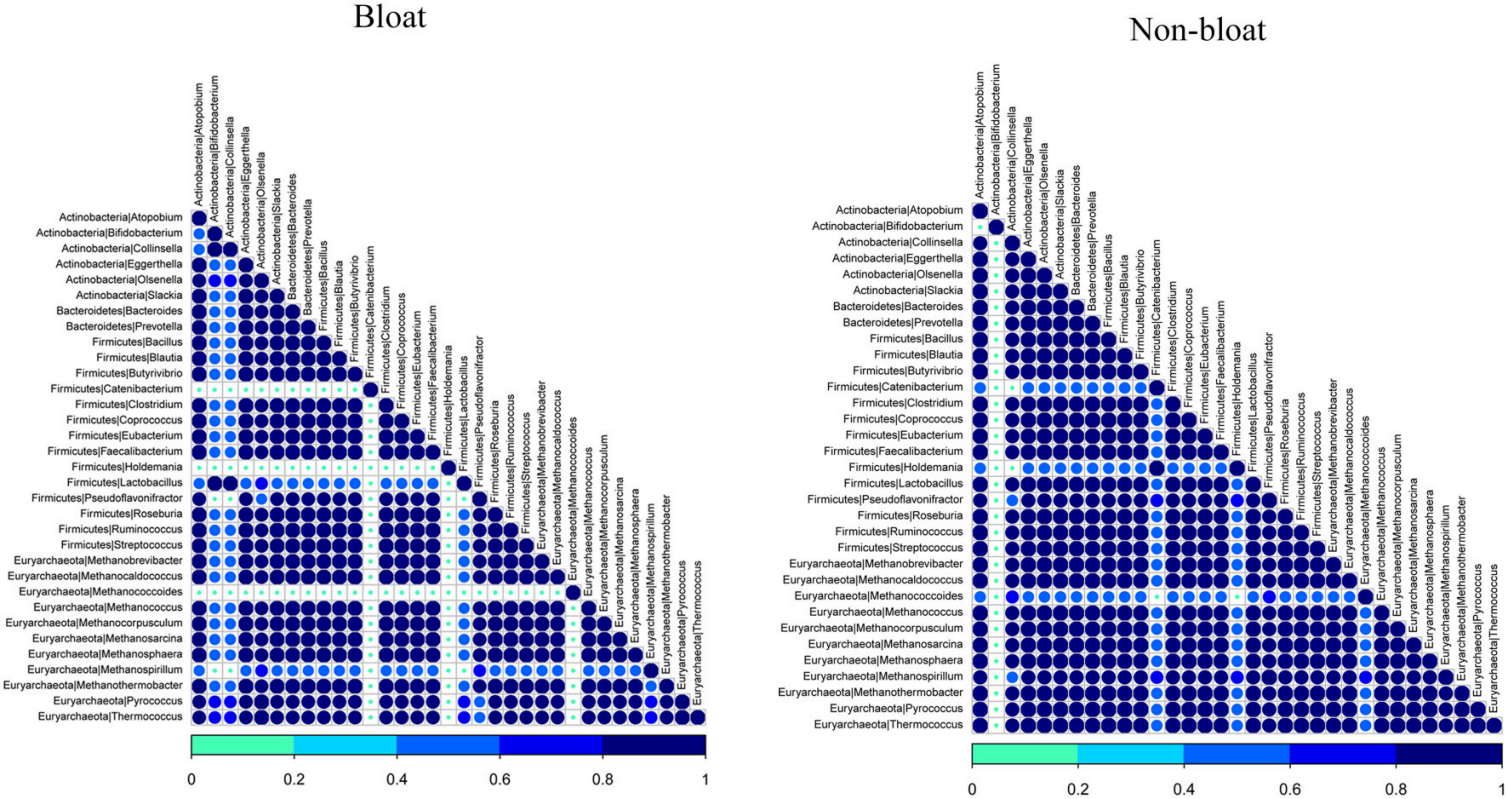
**Analysis of co-occurrence among the most abundant bacterial and archaeal lineages scored using the Dice index for bloated and non-bloated rumen samples respectively**. Co-occurrence is shown by the color code (navy blue, high co-occurrence; sky blue, moderate co-occurrence; green, low co-occurrence) at the bottom.

As rumen microbes work cohesively to perform various metabolic activities in the rumen, we sought to determine the associative interactions between bacteria and archaea present in bloated and non-bloated samples using co-occurrence analysis based on the Dice index (Figure 2). For this analysis, we selected the most abundant genera (>0.01%) from both bacterial and archaeal communities. In total, we had six genera from Actinobacteria, two genera from Bacteroidetes and 14 genera from Firmicutes and 11 genera from Euryachaeota. Associations were presented for non-bloated and bloated samples (Figure 2). Co-occurrence is shown by the color code (navy blue, high co-occurrence; sky blue, moderate co-occurrence; green, low co-occurrence).

It is apparent that there are strong symbiotic interactions between a majority of bacterial and archaeal genera in the non-bloated samples. However, some interactions fade out in bloated samples. An exception, *Bifidobacterium* was observed to co-occur with most of bacterial and archaeal genera in bloated samples while such co-occurrence was minimal in non-bloated samples. On the contrary, *Collinsella* from Actinobacteria, *Catenibacterium, Holdemania*, and *Lactobacillus* from Firmicutes and *Methanococcoides* and *Methanospirillum* from archaea were observed to weakly interact with all bacterial and archeal genera (selected for co-occurrence analysis) in bloated samples when compared to non-bloated samples in this study. These results reveal that associative patterns occur among both ruminal bacteria and archaea in steers maintained on wheat pasture which can be interrupted with abrupt changes in rumen microbial fermentation as in the case of frothy bloat.

### Metabolic potential

Using the Subsystems functional database, we identified genes associated with carbohydrate and protein metabolism that accounted for 10% each, respectively of total gene content detected in this study (Table S8). No differences were noted in the major metabolic pathways, however, differences (*P* < 0.05) were noted in mineral metabolism such as sulfur and phosphorus and also in vitamin and prosthetic group metabolic pathways. At level 3, specific genes associated with normal carbohydrate metabolism, particularly glucose, appears to be higher in non-bloated samples while genes responsible for methane formation and activity of archaea were more pronounced in bloated samples (Table S9).

### Changes in gene content with incidence of bloat

To describe the functional contribution of the dominant bacterial phyla we compared changes in gene content between bloated and non-bloated samples. Odds ratios were calculated for both pairs and color coded to show changes in gene content (Figure S2). Gene sequences from Firmicutes and Proteobacteria were more abundant in bloated samples (odds ratio >1) and were found to have associations with all identified metabolic functions. Gene sequences that were higher in bloated samples were mostly assigned to Firmicutes which were involved with a number of carbohydrate metabolic pathways and also observed to participate in other functions such as amino acid metabolism, energy metabolism, lipid metabolism, and secondary compound metabolism.

### Carbohydrate active enzymes (CAZymes)

**Figure 3:**
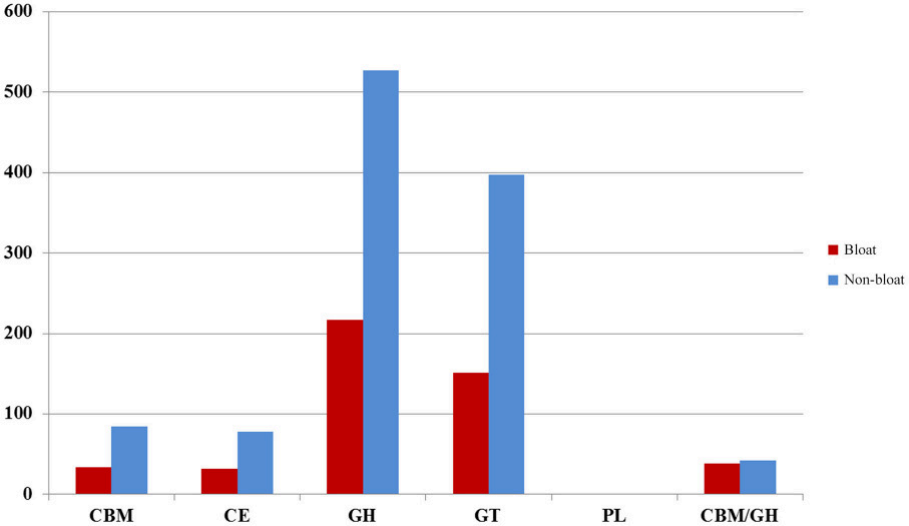
**Distribution of CAZy families across bloated and non-bloated samples**. CBM, Carbohydrate-Binding Module; CE, Carbohydrate Esterase; GH, Glycoside Hydrolase; PL, Polysaccharide Lyases.

As carbohydrate metabolism was found to be one of the most significant metabolic activities in the current study, we sought to assess the potential of lignocellulose breakdown by identifying putative carbohydrate-active gene sequences from the six metagenomic libraries. A minimum of 47,000 putative genes per each sample were identified in this study by MetaGeneMark which were further aligned to selected CAZy families. We were able to detect CBM/GH/CE/GT/PL families that were active in lignocellulose breakdown, however, GH and GT families were well represented (>200 numbers) while the other three constituted <100 across all samples (Figure 3). Notably, all these families were much lower in number in bloated samples when compared to non-bloated samples. The putative GH families (Table 5) were grouped into four categories (cellulases, endohemicellulases, debranching enzyme, and oligosaccharide degrading enzymes) based on their function. We found an abundance of oligosaccharide degrading enzymes (>75%) followed by endo-hemicellulases (14–20%) and debranching enzymes (4–6%) and cellulases (3%) across all samples. Interestingly, we found that all these GH enzymes were nearly halved in bloated samples indicating an impairment of carbohydrate metabolism.

**Table 5:** **Comparison of the CAZyme genes detected in bloated and non-bloated rumen contents**.

**Cazy family**	**Cellulases**		**Cazy family**	**Endo-hemicellulases**		**Cazy family**	**Oligosaccharide-degrading enzymes**		**Cazy family**	**Debranching enzymes**	
	**Bloated**	**Non-bloated**		**Bloated**	**Non-bloated**		**Bloated**	**Non-bloated**		**Bloated**	**Non-bloated**
GH5	4	13	GH8	0	2	GH1	14	48	GH77	6	13
GH45	0	1	GH9	4	6	GH2	11	31	GH78	5	3
GH87	0	1	GH10	0	5	GH3	26	62			
			GH12	0	3	GH4	8	8			
			GH19	0	1	GH13	29	53			
			GH27	5	1	GH31	1	11			
			GH28	0	4	GH35	0	6			
			GH29	1	2	GH39	6	16			
			GH30	0	6	GH42	1	1			
			GH47	1	1	GH43	5	13			
			GH51	0	8	GH65	1	2			
			GH53	9	26	GH92	1	3			
			GH99	1	6	GH94	12	15			
			GH115	0	4	GH95	1	4			
			GH120	0	2	GH97	0	11			

## Discussion

Frothy bloat is a complex metabolic disorder mitigated by interactions among environmental, plant, and animal factors. Bloated rumen contents had higher pH (6.1 vs. 5.8) and twice the value of viscosity (4 vs. 2) when compared to non-bloated contents, indicating a disruption of normal fermentation pattern. As recent evidences indicate that the composition of rumen microbes is directly related to the health and production performance of cattle (McCann et al., 2014; Morgavi et al., 2015), we sought to investigate changes in the microbial ecology and functional potential of the rumen microbiome and fermentation pattern in the rumen of stocker cattle that succumbed to bloat.

### Changes in the rumen microbiome with bloat incidence

A significant finding from this study was occurrence of frothy bloat was not associated with wholesale shifts in microbial populations but significant tradeoffs among lineages within bacterial and archeal phyla. For example, in the bloated samples, *Bacteroides* and *Parabacteroides* genera increased while *Prevotella* decreased in Bacteroidetes phylum. Similarly, *Eubacterium, Clostridium*, and several other genera increased while *Ruminococcus* and *Lactobacillus* decreased in Firmicutes in the bloated samples. *Methanobrevibacter* alone increased while several other genera in the Euryarchaeota phylum decreased in bloated samples. These data indicate that frothy bloat in stocker cattle grazing wheat pasture is not associated with specific microbe(s) but due to several changes in the community structure across all microbial domains. In this study, we were able to investigate changes and interactions among bacteria and archaea using a metagenomics approach despite the limited sample size and depth of sequencing. A more in-depth sequencing and/or complemented with 18S/ITS region targeted amplicon sequencing among bloated and non-bloated samples is certainly warranted to glean shifts across all microbial domains including ruminal protozoa and fungi.

There appears to be pronounced synergistic relationships between bacteria, particularly Firmicutes, and methanogens in non-bloated samples, according to our co-occurrence analysis which is similar to the findings of Kumar et al. (2015). Such complex interactions were disrupted in bloated samples where *Lactobacillus, Haldemania, Collinsella*, and *Catenibacterium* do not seem to co-occur with other bacteria and methanogens (Figure 2). Similarly, *Methanococcoides* and *Methanospirillum* showed weaker interactions with other bacteria and archaea suggesting their dependency on other bacteria and/or archaea is decreased in bloated samples.

*Bifidobacterium* is the only genus to exhibit interactions with all genera in bloated samples. This interaction is more pronounced with *Lactobacillus* and *Collinsella*, which appear to exhibit less co-occurrence with other genera in bloated samples compared to non-bloated samples. The significance of *Bifidobacterium* and *Lactobacillus* in the lower gut is well established in humans and these two bacteria have been used as probiotics to treat several gastrointestinal disorders in humans (Moon et al., 2015) and animals (Malmuthuge et al., 2015). However, the role of *Bifidobacterium* in the rumen is less significant probably due to its lower abundance. This genus has been classified as a starch digestor (Xia et al., 2015). Recently, it has been shown that indigestible carbohydrates such as linear arabino oligosaccharides and linear arabinans increased the growth rates of *Bifidobacterium* from 6.3 to 15% in *in vitro* batch fermentation using human feces, indicating that *Bifidobacterium* has access to these indigestible carbohydrates (Moon et al., 2015). These evidences enabled us to speculate that *Bifidobacterium* is possibly one of the primary bacterium involved with the digestion of polysaccharides that are trapped in the biofilm and substrates that are released during the breakdown process by *Bifidobacterium* are being utilized by other bacteria, thus leading to the observed co-occurrence patterns with several other genera.

The low per cent of sequences annotated to functional assignment precludes us from making any focused correlations associating rumen microbes with metabolic pathways. Nevertheless, we have identified a majority of gene sequences from Firmicutes that were relatively more abundant in bloated samples when compared to non-bloated samples (Figure S2). Genera such as *Lachnoclostridium, Eubacterium, Blautia*, and *Butyrivibrio* appear to participate in a majority of metabolic pathways indicating their role in the event of bloat with a higher number of sequences contributing to carbohydrate metabolic pathways. It was reported earlier that genera including *Streptoccocus bovis, Bifidobacterium* spp., *Borrelia* sp., *Butyrivibrio* spp., *Prevotella* spp.*, Eubacterium ruminantium, Ruminobacter amylophilus, Ruminococcus bromii, Succinimonas amylolytica*, and *Lactobacillus* sp. were considered as starch fermenting bacteria, however, these genera were also able to derive energy from complex carbohydrates other than starch (Xia et al., 2015). Our data indicate *Ruminococcus, Lactobacillus, Collinsella*, and *Prevotella* to be active in non-bloat samples suggesting that oligosaccharides present in wheat pasture were able to meet the requirements of these bacteria. Rapid fermentation of oligosaccharides in the rumen is also evident by the diversity and abundance (70%) of oligosaccharide breaking enzymes in non-bloated samples. However, as fermentation is disrupted with biofilm formation, oligosaccharides necessary for the growth of the above mentioned bacteria are possibly trapped in the biofilm matrix. In contrast, other bacteria such as *Clostridium, Eubacterium, Butyrivibrio*, and several other bacteria were able to proliferate suggesting that these bacteria can utilize the oligosaccharides trapped in the biofilm or grow utilizing other available substrates. Further, the decrease in diversity and abundance of oligosaccharide breaking enzymes and hemicellulases support our conclusion that oligosaccharides breakdown is impaired owing to the formation of biofilm in bloated samples. Interestingly, *Methanobrevibacter* appears to be more resilient to bacterial shifts and their associated fermentation pattern utilizing a majority of the hydrogen released to make methane, while the abundance of genera such as *Methanococcoides* and *Methanospirillum* was found to be influenced by host effects (Zhou et al., 2012) and were not sustainable in the presence of biofilm as observed in this study.

Both 16S and shotgun sequencing data revealed the predominance of Firmicutes and Bacteroidetes in steers grazing wheat pasture, similar to other reports on the rumen microbiome (Mohammed et al., 2014; Lima et al., 2015). However, shotgun metagenomics recovered Actinobacteria and Proteobacteria in addition to Firmicutes and Bacteroidetes. Bloated rumen contents showed a higher abundance of Firmicutes and Proteobacteria while Bacteroidetes and Actinobacteria were reduced. Recent evidence indicates Proteobacteria appear to become co-dominant in ruminants fed starch-based diets (Pope et al., 2011; Kang et al., 2013; Petri et al., 2013). In general, the recovery of Proteobacteria is lower in 16S based studies as opposed to metagenomic studies. In a different study (Pitta et al., 2016), we found the recovery of Proteobacteria to be much higher with shotgun metagenomics compared to 16S based bacterial diversity in the rumen of dairy cows. Further, we demonstrated that Proteobacteria were actively involved with several metabolic pathways in the rumen. It was reported that although the relative abundance of Proteobacteria was smaller compared to other bacterial phyla in steers maintained on grain diet, they play a major role in the rumen metabolism (Kang et al., 2013). In this study, the abundance of Proteobacteria was much smaller in 16S data while the contribution was noticeable in the shotgun metagenomic data. Proteobacteria in this study were comprised of several genera whose individual abundance is <0.2%. However, a majority of these genera were more abundant in bloated samples suggesting that they are involved in biofilm formation, biofilm fermentation, and/or soluble carbohydrate digestion. Further studies are required to investigate the functional role of ruminal Proteobacteria in stocker cattle on wheat pasture and involvement in frothy bloat occurrence in grazing cattle. Future studies should include application of metatranscriptomics to complement metagenomics which may reveal the functionality of these changes in microbiota. Such information would support development of feed additives or supplements that have the potential to favor the growth of desired commensal bacteria and decrease the accumulation of biofilm which ultimately reduce the incidence of frothy bloat.

An inherent limitation in this study is the low number of sequences assigned to specific metabolic pathways. Although gene sequences assigned to carbohydrate and protein metabolism did not differ between the rumen samples of bloated and non-bloated samples at level 1 classification of Subsytems database available on MG-Rast Server, we did observe that non-bloated samples had numerically higher numbers of gene sequences associated with polysaccharide and monosaccharide breakdown when compared to bloated samples at level 2 and level 3 (Table S8) suggesting differences were noticeable at the enzyme level. Further, a snapshot view of CAZymes identified in this study clearly illustrates that enzymes responsible for normal fermentation of polysaccharides and oligosaccharides except for GH27 were reduced by several fold in bloated samples compared to non-bloated rumen samples indicating a depression of normal fermentation processes. The CAZyme GH27 (α-galactosidases) are specific to cleave glycosidic linkages in complexed oligosaccharides or lipids in lysosomes (Willems et al., 2014). An increase in GH27 in bloated samples may likely be to hydrolyze oligosaccharide linkages locked in the biofilm. Overall, perturbation in fermentation processes are the consequences of less metabolically active bacteria and probably a lower microbial population density, as we reported a reduction in the rumen bacterial diversity in steers grazing wheat pastures during the bloat prone period (Pitta et al., 2014a). Taken together, alterations in microbial populations can interfere with the normal metabolic fermentation patterns in the rumen, as observed in bloated rumen samples, thus reinforcing our hypothesis that bloat occurrence is the consequence of altered microbial activity. We envision that more in depth sequencing of bloated samples, coupled with a more intensive survey of metabolically active bacteria through RNA targeted metatranscriptomics, can shed light on the host-microbes interactions in the event of bloat. Further, complete characterization of microbiota associated with biofilm at varying time points is necessary to understand the microbial interactions that may be associated with incidence of bloat.

## Conclusions

In summary, frothy bloat in stocker cattle grazing wheat pastures is associated with changes in both ruminal microbial and fermentation pattern. Wheat pasture induced frothy bloat is not associated with wholesale shifts in microbial populations but significant tradeoffs among lineages within each bacterial and archeal phyla. These microbial changes are accompanied by changes in microbial genes and enzymes resulting in perturbations in the normal fermentation processes. We speculate that certain clans of bacteria accelerate the formation of biofilm which further reduces the availability of fermentable substrates to support microbial growth, thus substantially altering microbial activity and ultimately resulting in bloat.

## Author contributions

WP and DP designed experiments; DP and JF executed experiments; NI, RS, and DP analyzed the data. DP, NI, and WP contributed to the paper, with input from all authors. All authors read and approved the final manuscript.

## Funding

Funding for this study was partly contributed by internal grants from Center for Host-Microbe interactions, University Research Foundation from University of Pennsylvania.

### Conflict of interest statement

The authors declare that the research was conducted in the absence of any commercial or financial relationships that could be construed as a potential conflict of interest.

## Supplementary material

Table S1 **16S rDNA based relative abundance (in percentage) for bacterial populations at the phylum level for each sample**.

Table S2 **16S rDNA based relative abundance (in percentage) for bacterial populations at the genus level for each sample**.

Table S3 **16S rDNA based bacterial OTUs that were significantly different between bloated and non-bloated rumen samples**.

Table S4 **Mean and significant difference for microbial domains between bloat and non-bloated samples as identified from shotgun metagenomic data**.

Table S5 **Mean and significant difference for bacterial phyla between bloat and non-bloated samples as identified from shotgun metagenomic data**.

Table S6 **Mean and significant difference for bacterial genera between bloat and non-bloated samples as identified from shotgun metagenomic data**.

Table S7 **Mean and significant difference for archaeal genera between bloat and non-bloated samples as identified from shotgun metagenomic data**.

Table S8 **Mean and significant difference of the Subsystems functional pathways (level 1) between bloat and non-bloated samples**.

Table S9 **Mean and significant difference of the Subsystems functional pathways (level 3) between bloat and non-bloated samples**.

Figure S1 **The most abundant bacterial phyla retrieved from the 16S rDNA data from each bloated and non-bloated samples**.

Figure S2 **Functional metagenomic analysis of the bacterial community**. The bacterial genera are represented at the bottom and KEGG functional categories at the right of the Figure. Higher the Odds ratio, higher the gene content in bloated samples. The colored tiles indicate the changes in gene content within each phylum between the bloated and non-bloated rumen contents. Color coded on the functional categories in the legend at the right indicates as follows: Yellow, Amino acid metabolism; blue, Carbohydrate metabolism; green, Energy metabolism; red, Lipid metabolism; dark green, metabolism of cofactors and vitamins; indigo, metabolism of terpenoids and polyketides; pink, Nucleotide metabolism; and brown, Xenobiotics biodegradation and metabolism.
