# Supplementary figures and images for: Metagenomic Analysis of the Rumen Microbiome of Steers with Wheat-Induced Frothy Bloat
Source: Front Microbiol. 2016 May 11;7:689. doi: 10.3389/fmicb.2016.00689 (PMC4863135; doi:10.3389/fmicb.2016.00689)

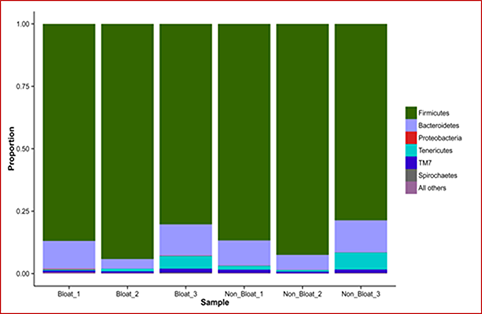

Supplement: Figure S1 — The most abundant bacterial phyla retrieved from the 16S rDNA data from each bloated and non-bloated samples. [file Image1.TIF]

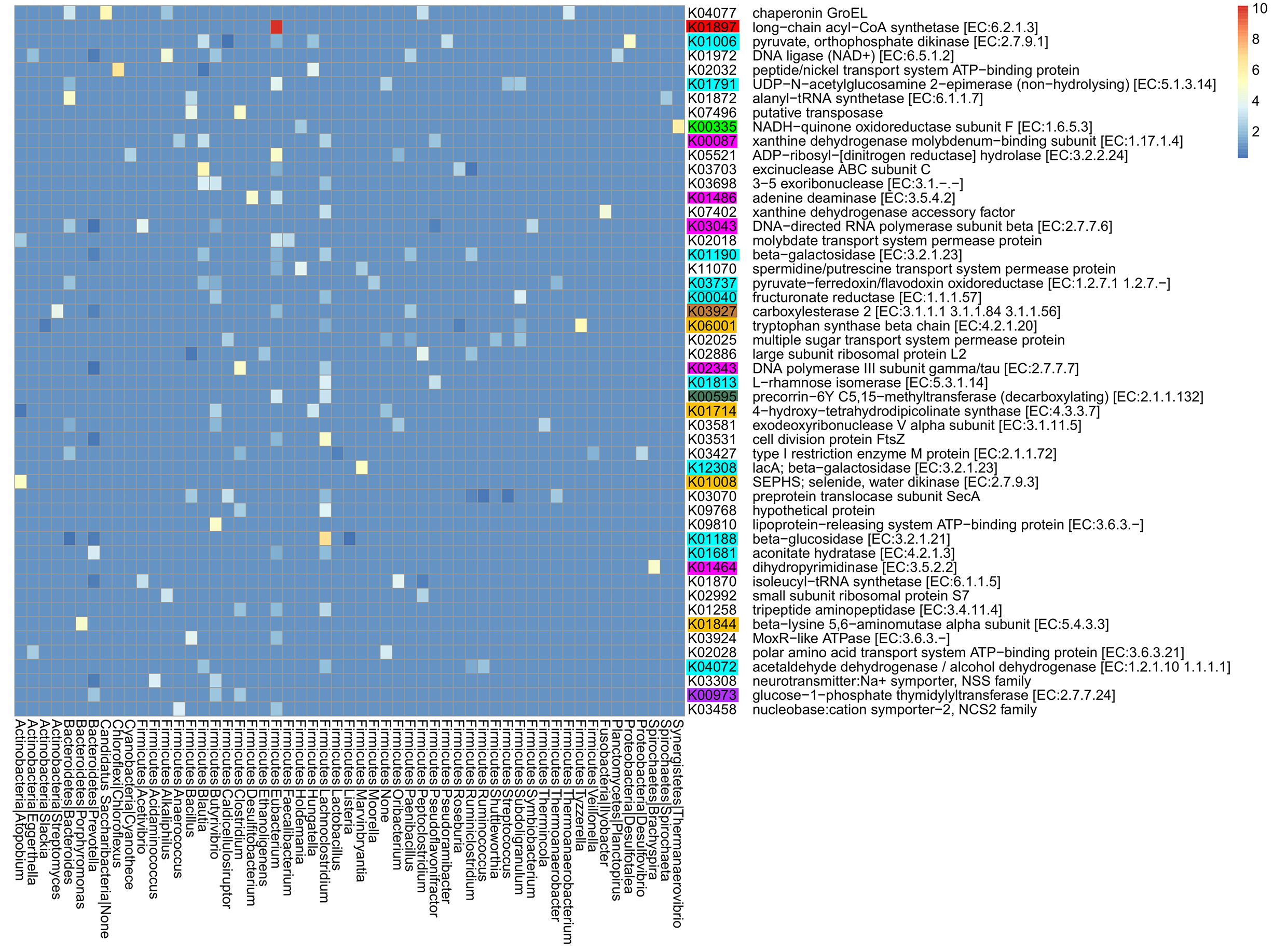

Supplement: Figure S2 — Functional metagenomic analysis of the bacterial community. The bacterial genera are represented at the bottom and KEGG functional categories at the right of the Figure. Higher the Odds ratio, higher the gene content in bloated samples. The colored tiles indicate the changes in gene content within each phylum between the bloated and non-bloated rumen contents. Color coded on the functional categories in the legend at the right indicates as follows: Yellow, Amino acid metabolism; blue, Carbohydrate metabolism; green, Energy metabolism; red, Lipid metabolism; dark green, metabolism of cofactors and vitamins; indigo, metabolism of terpenoids and polyketides; pink, Nucleotide metabolism; and brown, Xenobiotics biodegradation and metabolism. [file Image2.TIF]
